# UnSplicer: mapping spliced RNA-seq reads in compact genomes and filtering noisy splicing

**DOI:** 10.1093/nar/gkt1141

**Published:** 2013-11-19

**Authors:** Paul D. Burns, Yang Li, Jian Ma, Mark Borodovsky

**Affiliations:** ^1^Joint Georgia Tech and Emory Wallace H. Coulter Department of Biomedical Engineering, Atlanta, GA 30332, USA, ^2^Department of Bioengineering, University of Illinois at Urbana-Champaign, IL 61801, USA, ^3^Institute for Genomic Biology, University of Illinois at Urbana-Champaign, IL 61801, USA, ^4^School of Computational Science & Engineering, Georgia Tech, Atlanta, GA 30332, USA and ^5^Department of Bioinformatics, Moscow Institute of Physics and Technology, Moscow, 141700, Russia

## Abstract

Accurate mapping of spliced RNA-Seq reads to genomic DNA has been known as a challenging problem. Despite significant efforts invested in developing efficient algorithms, with the human genome as a primary focus, the best solution is still not known. A recently introduced tool, TrueSight, has demonstrated better performance compared with earlier developed algorithms such as TopHat and MapSplice. To improve detection of splice junctions, TrueSight uses information on statistical patterns of nucleotide ordering in intronic and exonic DNA. This line of research led to yet another new algorithm, UnSplicer, designed for eukaryotic species with compact genomes where functional alternative splicing is likely to be dominated by splicing noise. Genome-specific parameters of the new algorithm are generated by GeneMark-ES, an *ab initio* gene prediction algorithm based on unsupervised training. UnSplicer shares several components with TrueSight; the difference lies in the training strategy and the classification algorithm. We tested UnSplicer on RNA-Seq data sets of *Arabidopsis thaliana*, *Caenorhabditis elegans*, *Cryptococcus neoformans* and *Drosophila melanogaster*. We have shown that splice junctions inferred by UnSplicer are in better agreement with knowledge accumulated on these well-studied genomes than predictions made by earlier developed tools.

## INTRODUCTION

Vast volumes of RNA-Seq reads generated by next generation sequencing (NGS) technologies carry valuable information on patterns of gene expression. Still, to extract this knowledge from sequence data, RNA-Seq reads must be aligned to the reference genome. Many software tools have been developed in recent years to solve this problem. Notably, the problem is 2-fold because alignment can be ungapped (full-length read alignment) and gapped [alignment of a read spanning one or more initially unknown splice junctions (SJs)]. The term ‘splice junction’ refers to two adjacent ribonucleotides along RNA that were made adjacent by the RNA splicing and intron removing reaction. Identification of SJs (and thus the corresponding introns in genomic DNA) is critical for reconstructing exon–intron structures of eukaryotic genes.

A significant reduction in the ungapped alignment computational complexity was reached by application of the Burrows-Wheeler Transform in the software tools such as BWA (1,2), SOAP (3) and Bowtie (4). Existing software tools that map RNA-Seq reads to genomic DNA, both in ungapped and gapped fashions, include TopHat (5,6), MapSplice (7), SpliceMap (8), GSNAP (9), SOAPsplice (10), PASSion (11) and TrueSight (12). TopHat uses information from RNA-Seq reads mapped without gaps to derive exon (and intron) boundaries. MapSplice uses an ‘anchor and extend’ approach for mapping RNA-Seq read segments situated near and over SJs. Iterative remapping of whole RNA-Seq reads or read segments in parallel with SJ inference as well as filtering of false positives (FPs) in post processing are the hallmarks of the most recently developed tools. For instance, to eliminate incorrect alignments PASSion, SOAPsplice and TrueSight use paired-end information. Still, it appears that all tools existing to date make novel SJ predictions at a significantly higher rate compared with tools designed to align long Expressed Sequence Tag (EST) sequences such as BLAT (13) and GMAP (14).

Besides *bona fide* novel SJs that occur in functional transcripts, a significant fraction of novel SJ inferred by computational tools could be erroneous in some sense. Two major sources of inferring erroneous SJs are (i) suboptimal alignment methods and (ii) noisy splicing (15–17). Recent research indicates that many instances of novel SJs are manifestations of biochemical noise influencing the splicing process (15–20). Because some introns are spliced owing to random selection of one or both splice sites, such transcripts are likely to be functionally irrelevant. Importantly, it was shown that patterns of occurrence of experimentally determined novel introns can be closely matched by appropriate models of random splicing errors (15). Also, many novel splice sites have little evidence of evolutionary conservation across species (16), which, in fact, alone does not necessarily imply absence of biological function in the novel transcripts. Nonetheless, other observations show that the majority of functional transcripts are translated into proteins (21), as well as that there is one dominant functional transcript per gene for the majority of genes (22).

These observations allow the following statements: (i) the majority of functional transcripts annotated in genomes of model organisms are likely to be translated; (ii) the majority of functional transcripts carry nucleotide frequency patterns developed during evolution of protein-coding genes; and (iii) annotated exon–intron structures should play an important role in assessment of accuracy of inference of SJs from RNA-Seq data.

Also one could argue that the relevance of statements (i)–(iii) is higher for more compact eukaryotic genomes that have on average relatively short intergenic regions and thus a lower proportion of long noncoding RNAs. In this context, we will refer to eukaryotic genomes with length <500 Mb as compact genomes. We foresee the technique described below will apply primarily to transcriptomes and genomes of species with compact genomes; it is important to note that the vast majority of species whose genome and transcriptome sequences are available to date have compact genomes.

Some DNA sequence patterns developed in evolution at the exon–intron borders of functional genes have been used in earlier developed algorithms. For instance, some of them require the presence of intron terminal dinucleotides: canonical GT–AG pairs or semi-canonical GC–AG or AT–AC pairs. To delineate introns, the PASSion program (11) uses a sequence ‘pattern growth’ algorithm. Another recent program, TrueSight (12), assesses sequence patterns by using the Markov chain models of splice sites as well as of protein-coding and noncoding sequences. From a set of initially identified SJs, TrueSight selects a subset of the most confident ones as a data set for estimation of parameters of the Markov models. Classification of all other SJ candidates is made by logistic regression with parameters derived by the iterative expectation maximization (EM) algorithm.

In analysis of novel compact genomes and proteomes, we see further opportunity to add more information currently concealed in DNA sequence to algorithms of RNA-Seq alignment. First, parameters of sequence models can be obtained by effective self-training methods. Second, information on locations of protein-coding exons predicted by the self-training algorithms should help reduce rates of false-positive SJ predictions. Particularly, parameters of the sequence models can be determined by the *ab initio* eukaryotic gene finder GeneMark-ES (23,24). This algorithm derives its parameters from as yet unannotated genomic sequence via self-training, thus skipping the time-consuming step of training sets preparation. GeneMark-ES finds parameters of a hidden semi-Markov model (HSMM) that includes models of splice sites, the Markov models of coding and noncoding regions, intron and exon length distributions, etc. GeneMark-ES works effectively for eukaryotic genomes with homogeneous GC composition, which is a typical feature of compact genomes, though many large genomes with homogeneous GC composition are known as well. A new version of GeneMark-ES for genomes with inhomogeneous GC composition is now under development (Lomsadze, Borodovsky, personal communication).

Presented here is the new algorithm and software tool UnSplicer, related to the earlier developed TrueSight. UnSplicer combines the DNA sequence features with features derived from intron sequences delineated by RNA-Seq gapped alignments. UnSplicer uses GeneMark-ES to derive parameters of DNA sequence models. UnSplicer uses a total of nine features to discriminate functional SJs from spurious SJs generated by mapping errors or noisy splicing. While most RNA-Seq mapping programs accurately map ‘simulated’ reads to compact genomes, when confronted with ‘real’ RNA-seq data these programs produce a large number of novel and likely functionally irrelevant SJ predictions. UnSplicer, similarly to TrueSight, assigns a probabilistic score to each SJ prediction. As such, the score facilitates the ranking of predicted SJs, and the formation of a larger or smaller final set of predictions, depending on the operator defined score threshold. To assess the accuracy of UnSplicer and several other programs in mapping RNA-Seq reads to genomes we used RNA-seq data sets for four compact genomes with reliable annotation: *Arabidopsis thaliana*, *Caenorhabditis elegans*, *Cryptococcus neoformans* and *Drosophila melanogaster*. These four genomes have manually curated annotation of genes and transcripts, which, similar to (16), we used as a standard of annotation of functional transcripts. We have shown that for one and the same number of predictions, UnSplicer inferred more SJs that match annotation than other programs, as well as short exons embedded in single reads, than the other state-of-the-art RNA-Seq reads alignment tools.

## MATERIALS AND METHODS

### Data sets

Sequence data used in the project include genomes of the plant *A. thaliana*, the round worm *C. elegans*, the insect *D. melanogaster*, the fungus *C. neoformans* as well as RNA-Seq data sets for the same species (Table 1). Additional sequence data, simulated RNA-Seq data sets, for *A. thaliana*, were generated by the program Maq (1).

**Table 1.** Description of RNA-Seq data and reference genomes used in accuracy assessment

Species	Data set	Read length (nt)	Number of pairs (millions)	Genome version	Annotation version
*A. thaliana*	SRR360205	76	20.9	TAIR10	TAIR10
*C. elegans*	SRR359066	101	12.2	Ce10	RefSeq, Ensembl
*D. melanogaster*	SRR042297	75	13.6	r5.42	R5.42
*C. neoformans*	SRR563164	101	5.7	Broad institute^a^	Broad institute^a^

^a^*Cryptococcus neoformans var. grubii* H99 Sequencing Project, Broad Institute of Harvard and MIT.

### RNA-Seq read alignments

**Figure 1.**
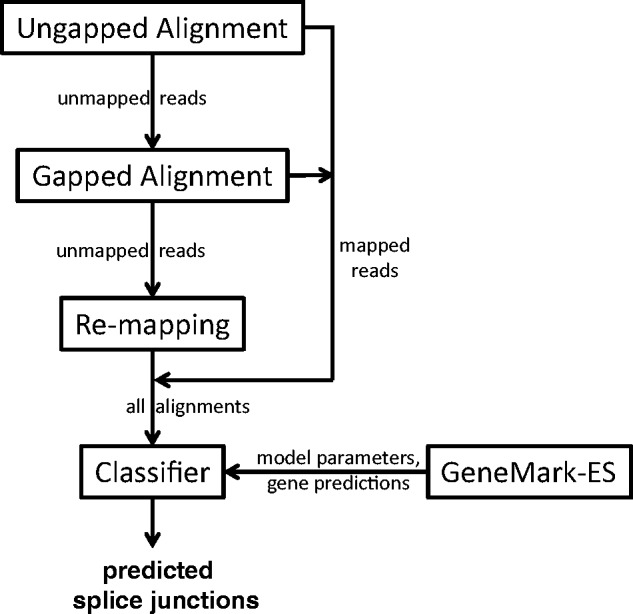
The UnSplicer algorithm diagram. First, RNA-Seq reads are attempted to be aligned to the reference genome without gaps (by Bowtie). Second, unmapped reads are attempted to be aligned with gaps by the ‘anchor-and-extend’ algorithm (the same as in TrueSight). Third, remaining unmapped reads are remapped to pseudo-transcripts reconstructed using predicted SJs (by Bowtie). Fourth, an SVM classifier with parameters derived by GeneMark-ES is used to assign a probabilistic score to each SJ candidate and those with scores higher than a chosen threshold are included in the final set of predictions.

Several read mapping algorithmic components of UnSplicer have been developed earlier: (i) the method of ungapped (full length) read alignment implemented in Bowtie, (4) and (ii) the ‘anchor and extend’ method of initial gapped alignment to genomic sequence implemented in TrueSight (12). A block diagram of the UnSplicer algorithm is shown in Figure 1.

After the first step—attempting ungapped alignment of all RNA reads to the genome by Bowtie—remaining unmapped reads are divided into short (18–25 nt) nonoverlapping segments and Bowtie is used again to attempt ungapped segment alignments. Failure to align a segment indicates that the segment may overlap a SJ. Such segments are subjects for the ‘anchor and extend’ alignment (12). Notably, ungapped alignments of the segments adjacent to the segment in question delimit the space in the genome where the potential intron should be situated. Therefore, the ‘anchor and extend’ alignment starts from the borders with fully aligned segments and continues into the interior of the unaligned segment with the goal to delineate intron(s) boundaries.

Two major factors complicate the ‘anchor and extend’ read alignment. First, fragments may not be aligned if they overlap two (or more) closely situated SJs. Second, the algorithm requires a minimum of 8 nt overhang over the SJ in the shorter side of alignment (to prevent incorrect alignments). The distance between two SJs, the exon length, should exceed the length of a segment (18–25 nt) for an anchor segment to be fully aligned inside. If the exon length exceeds 50 nt, then an anchor fragment will exist in any read that covers both SJs. Therefore, after the initial SJs are predicted, UnSplicer attempts to align unmapped reads to transcript sequences reconstructed around newly determined SJs. This realignment step is a common feature of other methods, such as PASSion and SOAPsplice.

In more detail, a transcript around a newly predicted SJ is reconstructed as a sequence of length *2L* (where *L* is the read length) by concatenating *L* nucleotides upstream and *L* nucleotides downstream from the already identified SJ positions in the reference genome. If other SJs (*n* of them) were predicted within *L* nt of the given SJ, then multiple transcripts were constructed. For each candidate SJ, a set of *2^n^* possible reference transcripts is formed (by splicing or not splicing each of *n* neighboring intron candidates). In practice, this approach may produce a large number of sequences if the read length is long (≥100 nt), and if there is a large number of false-positive SJ predictions. This number could be controlled by considering only SJs with intron lengths <10 000 nt; the vast majority of introns in compact genomes satisfy this requirement.

Note that for paired-end reads supposed to reside in opposite strands, spliced alignments that are ‘not’ in the opposite strand from a sibling’s alignment must be removed. This filtering step is also common for other methods, such as PASSion and SOAPsplice. Also, a position-dependent constraint must be applied to paired-end reads mapping: for example, a left-hand read mapped to a positive strand must have a lower coordinate than the right-hand read.

### Intron modeling

Another major task of UnSplicer, estimation of genome-specific parameters of the sequence models, is accomplished by GeneMark-ES, the *ab initio* gene-finding algorithm (23,24). GeneMark-ES estimates parameters of a HSMM of genomic sequence by iterative unsupervised training. The parameters are estimated before running the alignment pipeline. For compact genomes with homogeneous GC composition, GeneMark-ES often predicts intron boundaries with 90% or better sensitivity and specificity (23,24). In the HSMM, the splice sites hidden states emit fixed length nucleotide sequences with frequencies defined by position-specific frequency matrices (PSFMs); exon and intron hidden states emit variable length sequences described by Markov chain models. The donor PSFM spans 3 nt upstream of the intron 5′ end, and 6 nt downstream. The acceptor PSFM spans 20 nt upstream of the intron 3′ end, and 1 nt downstream. The PSFM models, treated as nonuniform Markov chains, can be of either zero or first order. Particularly, for all four genomes we worked with here, the donor and acceptor models are first order.

**Figure 2.**
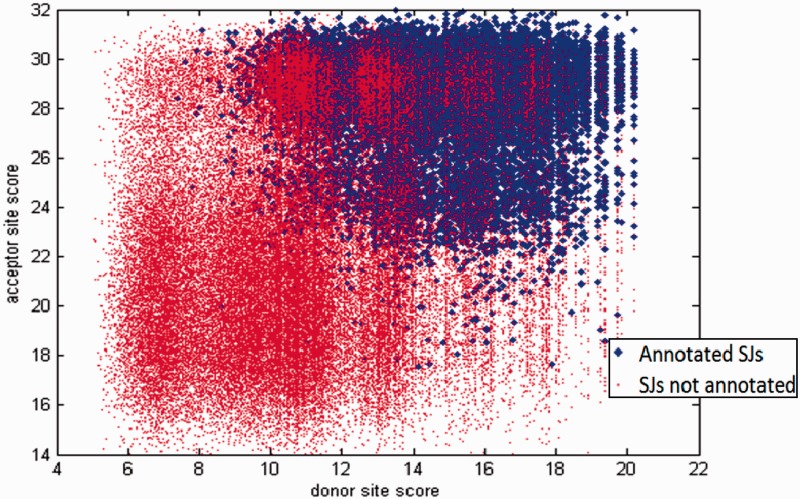
Each point in the scatter plot represents a SJ, defined by a gapped alignment of an RNA-Seq read (SRR360205 set) to genome of *A. thaliana*. The point coordinates are equal to the scores of the SJ’s donor and acceptor sites. The scores were computed with donor and acceptor model parameters determined by GeneMark-ES. The blue dots represent SJs annotated in TAIR10. Red dots correspond to mapped SJs not found in TAIR10 annotation.

To classify mapped SJs (and corresponding introns) UnSplicer uses nine features derived from two data sources: RNA-Seq read alignments and genomic sequence. Alignment-based features are as follows: (i) gapped (alignment) coverage skew, (ii) gapped alignment depth (the number of alignments confirming a SJ), (iii) gap (intron) length, (iv) entropy and (v) minimal read overhang length. While these five features were described in detail earlier (12), there are a few minor differences (for full definitions see Supplementary Materials). The sequence-based features are (vi) donor scores, (vii) acceptor scores, (viii) frameshift indicator and (ix) strand concordance indicator. The log likelihood ratio scores of candidate SJs are computed with use of parameters of splice site PSFMs. For a given intron, identified by a gapped alignment of a read, a pair of donor and acceptor site scores could be depicted by a vector in a plane. The set of such vectors corresponding to spliced alignments of the SRR360205 set of RNA-Seq reads to the *A. thaliana* genome is shown in Figure. 2. The vectors representing introns annotated in the TAIR 10 database of *A. thaliana* genome are shown by blue dots, while the vectors representing not-annotated introns are shown by red dots. The sets of blue and red dots are overlapping, however, there is a clear potential to use information on splice site scores for discrimination between annotated (likely correctly predicted) and not-annotated (likely erroneously predicted) introns.

The output of GeneMark-ES applied to a new genome includes both the estimates of algorithm parameters and the *ab initio* predicted genes. Information on locations of predicted genes is used in two additional SJ features defined by UnSplicer, the frameshift indicator and the strand concordance indicator. The frameshift indicator takes value 1 if (i) the predicted intron is situated between protein-coding exons and (ii) the reading frame shifts on splicing and does not fit the coding frame initially predicted by GeneMark-ES for the downstream exon. Otherwise, the frameshift indicator takes value 0. The second feature, the strand concordance indicator, takes value 1 if the mapped intron appears in the opposite strand of a predicted gene and value 0 otherwise (Supplementary Figure S4).

Three SJ features out of the 10 defined by TrueSight (12) were not included in the UnSplicer algorithm: the coding potential, the multiple mapping score and the number of alignment mismatches.

Coding potential was removed to improve sensitivity to SJs connecting noncoding exons. The multiple mapping score and the mismatch score were removed since we observed them to be less informative when mapping to compact genomes. The former is particularly useful for correctly mapping reads across long introns that are rare in compact genomes. The remaining seven SJ features of TrueSight were used in UnSplicer, along with the two new features described above (see also Supplementary Materials).

### Classification of SJ candidates

Each gapped alignment of a read, made in the first step of the UnSplicer run, generated a candidate SJ and intron to be assigned a probabilistic score by the classifier in the second step. Classification of candidate SJs (introns) was based on construction of a decision boundary in the six dimensional SJ feature space.

We used the radial basis function support vector machine (SVM) algorithm with Gaussian kernel (25) to classify the candidate SJs (introns). To determine the SVM parameters and decision boundary for each genome, we selected two nonoverlapping sets of SJs labeled as positive and negative examples, a training set and a development set. The training set is used to train the classifier with two parameters *c* and 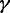
selected from a finite set of parameter pairs. The development set is used to evaluate the accuracy of each classifier in the trained set and select a pair of (*c*, 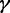) with best accuracy on the development set (see below).

We needed a proxy procedure that would generate the training set of labeled examples (true or false SJ) in the absence of correct genome annotation. Analysis of distributions of values ‘shorter overhang’, ‘entropy’ and ‘coverage skew’ in the initially detected SJs among annotated and nonannotated SJ gave an insight how to design such a proxy procedure.

**Figure 3.**
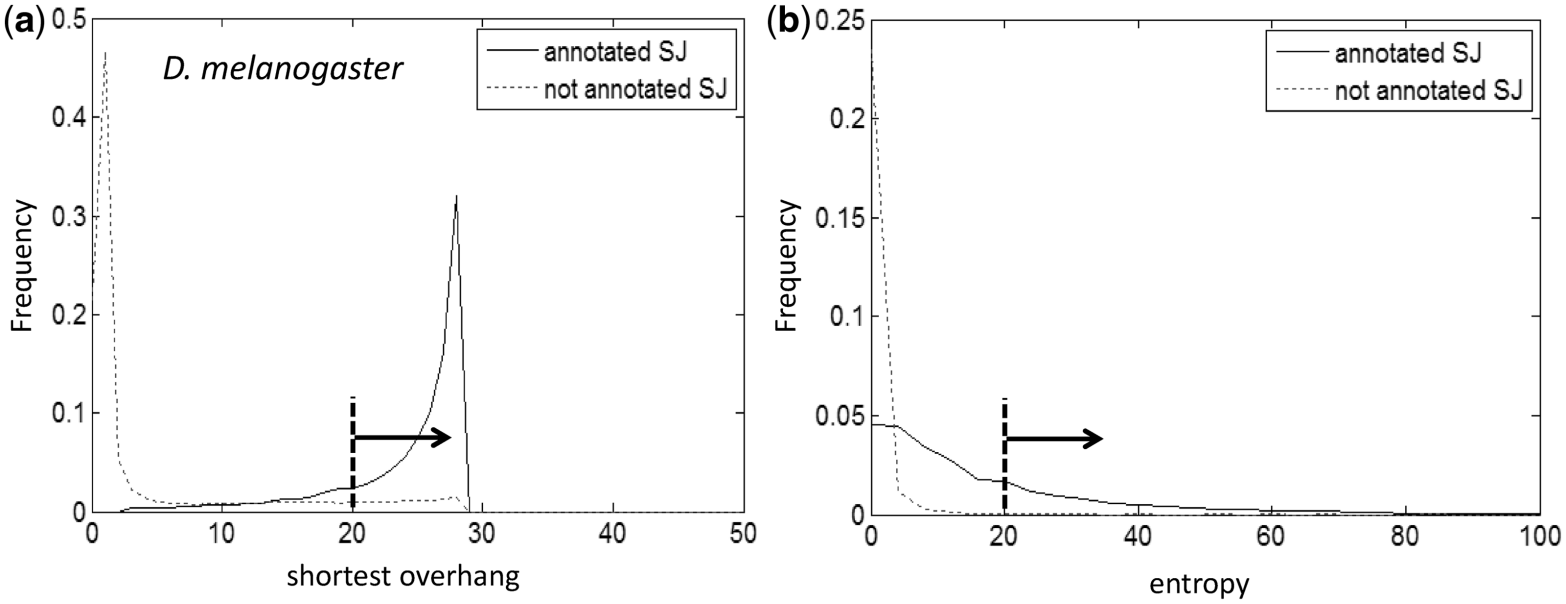
Histograms of shorter overhang values (**a**) and entropy scores (**b**) are shown for alignments of RNA-Seq reads (SRR042297 set) to the *D. melanogaster* genome. A set of positive examples was formed from read alignments having both a shorter overhang >20 nt and an entropy score >20, as indicated by arrows.

Positive examples in the training set were selected by sampling candidate SJs with long enough shorter overhang (>20 nt) complemented by a high value of entropy (>20). Distributions of values of these two variables for the set of SJs confirmed by annotation of *D. melanogaster* genome and the set of SJs not confirmed by annotation are shown in Figure 3. Notably, in the set of SJs selected as positive SJs for *D. melanogaster*, >98% were annotated in Flybase (ver. 5.42).

**Figure 4.**
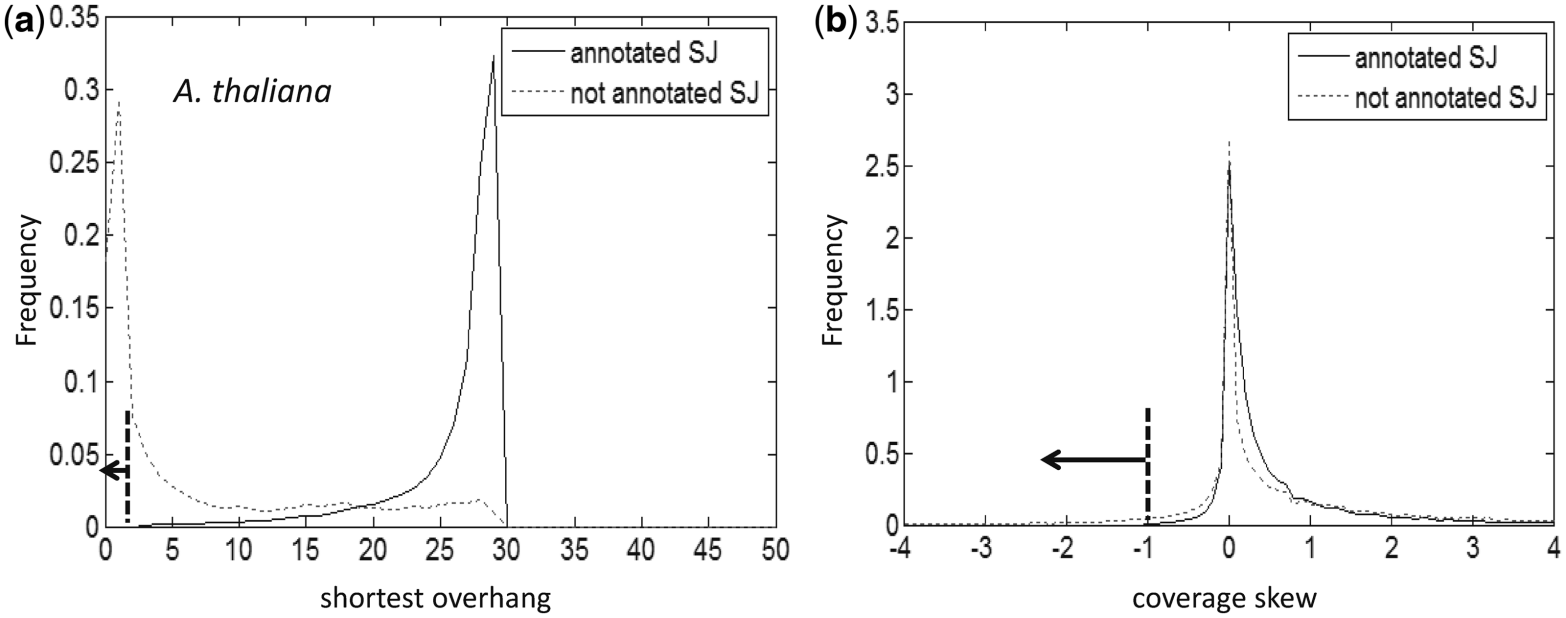
Histograms of shorter overhang values (**a**) and coverage skew scores (**b**) for gapped alignments of *A. thaliana* RNA-Seq reads (SRR360205 set). The set of predicted SJs with the shortest overhang value (<3) is highly enriched with negative examples. Similarly enriched with negative examples is the set of candidate SJs with coverage skew score <−1. SJs with scores situated in either of the regions indicated by arrows were labeled as negatives.

Negative examples in the training set were selected by sampling candidate SJs with ‘either’ a coverage skew score <−1, or a shorter overhang length of ≤2 nt. Distributions of shorter overhang length and coverage skew scores for the sets of confirmed and not confirmed SJs candidates obtained by mapping RNA-Seq reads from SRR360205 set to the genome of *A. thaliana* are shown in Figure 4. Among the SJs selected as a negative set, <1% were annotated in TAIR 10.

We used the three features, overhang, entropy and coverage skew, to select the training set of 10 000 SJs, equally divided between positive and negative examples. The other six features were used in the classification algorithm: donor and acceptor site scores, intron length, frameshift indicator, strand concordance indicator and gapped alignment depth.

For each genome, we also selected development sets with 5000 positive and 5000 negative examples not overlapping with the training set. The true or false label for each mapped intron in the development set was assigned with respect to a match of such an intron to one of the introns predicted by GeneMark-ES in the genomic sequence.

**Figure 5.**
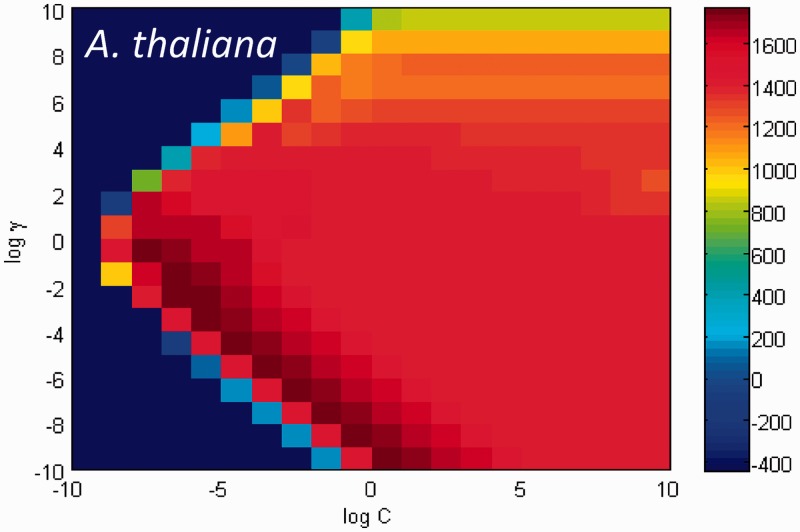
A heat map of values W = TP – FP produced by the SVM classifier with parameters 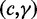 for the development set of *A. thaliana*. A grid search was performed to find the best kernel parameters *c* and 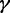 (where 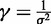). The optimal point with respect to W is 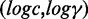
= (−7,−2).

The SVM Gaussian kernel has two parameters: the error cost (*c*) and width (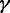). We needed to find the values of *c* and 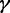 that achieved correct classification of the largest number of the mapped SJs. We used the LIBSVM package (26) to train the SVM on the training set, with parameters (*c*, 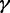) taken from a finite lattice. Because the training set cannot be used for fair evaluation of classification quality, we have the development set for this purpose. As the measure of quality of classification on the development set we chose the difference between the number of true- and false-positive predictions made by the SVM with given parameters. Each of the trained SVMs was used for classification of members of the development set and then, the SVM with parameters (c*, 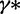), producing the least error of classification among all (*c*, 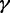) on the lattice, was selected. For instance, the results of classification made by the array of SVMs on development set for the *A. thaliana* genome could be visualized as a heat map on the (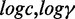) grid (Figure 5). The best values of (c*, 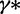) on the grid were [exp(−7), exp(−2)].

The SVM with genome-specific parameters (c*, 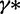) could be applied to classify the entire set of *N* candidate SJs, (e.g. 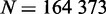 for the *A. thaliana* data set SRR360205). Still we have made one more step.

Output of the SVM can be mapped into posterior probabilities. A posterior probability value (a score) 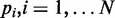 was assigned to a SJ with set of features 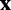 using the following method (27):



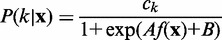

where 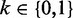 represents label class (1 is a ‘true’ SJ label, and 0 is ‘false’), 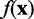
is the classification value for vector 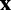 (+1 or −1, averaged over a 5-fold cross validation), 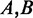 are constants found by maximizing the likelihood of the training data (27), and 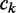 is a normalization constant chosen so that 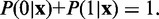


The probabilistic score assigned to each candidate SJ facilitates ‘flexibility’ of the classification procedure. The set of positive SJs could be identified as a subset of all candidate SJs with scores s > S, where S is the chosen score threshold. This set of newly predicted ‘positive’ SJs could be readily split into the SJs confirmed by the genome annotation (true positive, TP) and SJs not confirmed (FP). A full receiver operating characteristic (ROC) curve can be determined by variation of the threshold within the [0,1] interval; conversely, any point of the ROC curve can be chosen as an operating point for classification (see Figures in the ‘Results’ section). Note that normalization of the ROC curve limits to [0,1] intervals requires knowledge of total numbers of true and FPs in the set of objects (RNA-Seq reads with or without SJs) presented for the analysis.

## RESULTS

We assessed performance of UnSplicer (with Bowtie version 0.12.7) as well as four other RNA-Seq alignment programs: PASSion (v1.2), SOAPsplice (v1.9), TrueSight (v0.06) and TopHat2 (v2.0.8, with Bowtie version 2.1.0). We also attempted to assess performance of MapSplice (7) and GSNAP (9); however, in the runs of these programs we observed a significantly larger number of FPs; therefore, we do not cite these results. For benchmarking purposes, simulated RNA-Seq reads were used in addition to several real RNA-Seq data sets.

### Mapping RNA-Seq reads from real data sets

**Figure 6.**
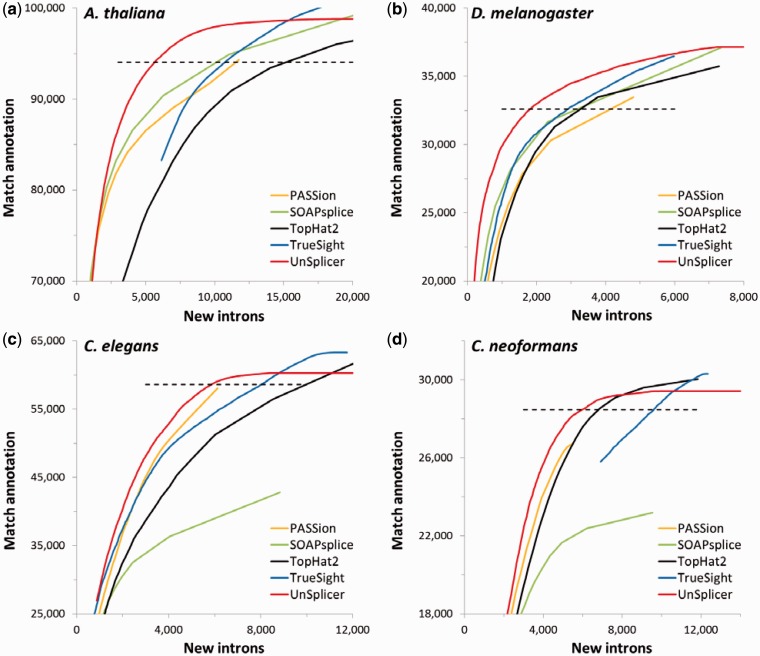
Performance of the five programs on real RNA-Seq data sets of *A. thaliana* (**a**), *D. melanogaster* (**b**), *C. elegans* (**c**) and *C. neoformans* (**d**). The curves represent nonnormalized ROC curves. The UnSplicer ROC curve shows higher performance, with fewer FPs for a given number of predicted SJs, except when TrueSight and TopHat2 show comparable performance in *C. elegans* (c) and *C. neoformans* (d) at the low value of the score threshold (in the zone with high number of novel introns not matching annotation).

To assess performance of RNA-Seq mapping programs on real data sets, we used RNA-Seq data available for *A. thaliana*, *C. elegans*, *D. melanogaster* and *C. neoformans* (Table 1). The introns (SJs) inferred by each program we divided into confirmed introns (matching previously annotated) and novel introns (not annotated) are illustrated in Figure 6. Because both UnSplicer and TrueSight compute probabilistic scores for each SJ, a given value of the score S (a threshold) selects a group of inferred SJs (introns) with score s > S. Division of the group into two numbers of novel (X) and confirmed (Y) introns produced a point on the curve, and the whole curve is produced when the threshold was varied from 0 to 1 with low parts of the curve corresponding to high thresholds and the top parts corresponding to the low threshold value. For the other three programs PASSion, SOAPsplice and TopHat2 we used SJ read coverage values as surrogate scores. The SJ coverage value threshold can be used to build the ROC curves for PASSion, SOAPsplice and TopHat2 (Figure 6a–d); the low parts of the curves correspond to the sets of SJ with high value of the coverage (high threshold), while the top parts correspond to the larger sets of SJ (low threshold). TopHat2 and UnSplicer in all the experiments described below had the max intron length restricted by 10 000, the limit covering the range of intron length in compact genomes. Interestingly, when TopHat2 intron length limit was changed to 500 000, it caused only a slight change in the results.

It was expected that the majority of those inferred SJs (introns) that were not confirmed by annotation in these well-annotated genomes were spurious, presumably originating from mapping errors or splicing noise (or both). Therefore, among two methods producing an equal number of introns confirmed by annotation, the one predicting fewer novel introns has better performance.

Using these criteria, UnSplicer performs better for all four species (Figure 6a–d) and especially for *D. melanogaster* and *A. thaliana*. Most of the time, for a given number of predicted introns that match annotation, UnSplicer made fewer predictions that do not match annotation than any other program. On the other hand, for a given number of predictions that do not match annotation, UnSplicer generated more predictions matching annotation than any other program. The dashed lines in Figure 6a–d correspond to the numbers of confirmed introns (TP) produced by UnSplicer with probabilistic score threshold 0.5. At this level of the threshold, the default operating point, UnSplicer generated from several hundred to several thousand fewer novel introns than the other programs, predicting the same number of introns confirmed by annotation. On the other hand, at the lowest values of the thresholds that release the highest numbers of novel introns into the inferred set of introns, TrueSight (for *C. elegans*) and TrueSight and TopHat2 (for *C. neoformans*) are able to find a higher number of TPs for the same number of FPs as UnSplicer (Figure 6c and d). While we can say that UnSplicer has shown the best performance in the tests for the four genomes, there is no consistent runner-up, nor is there a program that consistently underperformed in comparison with all others. Note that less impressive results produced by SOAPsplice for *C. elegans* and *C. neoformans* are likely to be caused by its default limit on the intron length (20 nt) not available for change as an input parameter. It is known that a large fraction of the introns in these two species are short (<20 nt).

Because of UnSplicer dependence on the ‘protein coding’ gene finder GeneMark-ES—several UnSplicer features used GeneMark-ES derived parameters, and also the development set for selection of the SVM parameters used introns predicted by GeneMark-ES—we specifically tested the ability of UnSplicer and other programs to detect SJs (introns) located in 5′ and 3′ untranslated regions (UTRs) and RNA genes (i.e. noncoding RNA regions) of one of the genomes we studied, *A. thaliana*. Introns inferred by each program were categorized as ‘coding’ or ‘noncoding’ with respect to the genome annotation (Supplementary Table S1). An intron was labeled as ‘noncoding’ only if exonic nucleotides adjacent to the intron were noncoding in all annotated isoforms. The ratios of ‘coding’ to ‘noncoding’ introns are shown in the bottom row of Supplementary Table S1. In general, the number of ‘coding’ introns is expected to be an order of magnitude higher than the number of ‘noncoding’ introns. We observed that PASSion produced the highest ratio among all programs except UnSplicer with threshold 0.9 (T = 0.9). As the UnSplicer threshold decreases, so decreases the abundance of the ‘coding’ type introns (see also Supplementary Figure S7). When the probability threshold decreases down to 0.1, the ratio of ‘coding’ to ‘noncoding’ introns detected by UnSplicer gets closer to ones detected by TrueSight, TopHat2 and SOAPsplice with default parameters.

The ‘anchor-and-extend’ technique facilitates finding short exons embedded into the RNA-Seq reads. The short exon detection performance was assessed using the RNA-seq data set SRR360205 with 76 nt long reads mapped to the *A. thaliana* genome. The numbers of short exons confirmed by annotation (likely TPs) and not confirmed by annotation (likely FPs) within eight length bins are shown in Supplementary Table S2. UnSplicer (T = 0.1) and TrueSight predicted a similar number of short exons 13 733 and 13 611, respectively. Overall, the specificity of UnSplicer in short exon prediction was higher than that of other programs, 92.8–96.1%, with TrueSight’s slightly lower at 92.1% (Supplementary Table S2). We attempted to analyze the performance of TopHat2 (with max intron length 10 000) in finding short exons. Because the short exons are not readily available in the TopHat2 output, we had to extract this information from the raw read mappings. We observed a slight increase in TP (by ∼200 in comparison with UnSplicer) with significant increase in FPs (data not shown).

**Figure 7.**
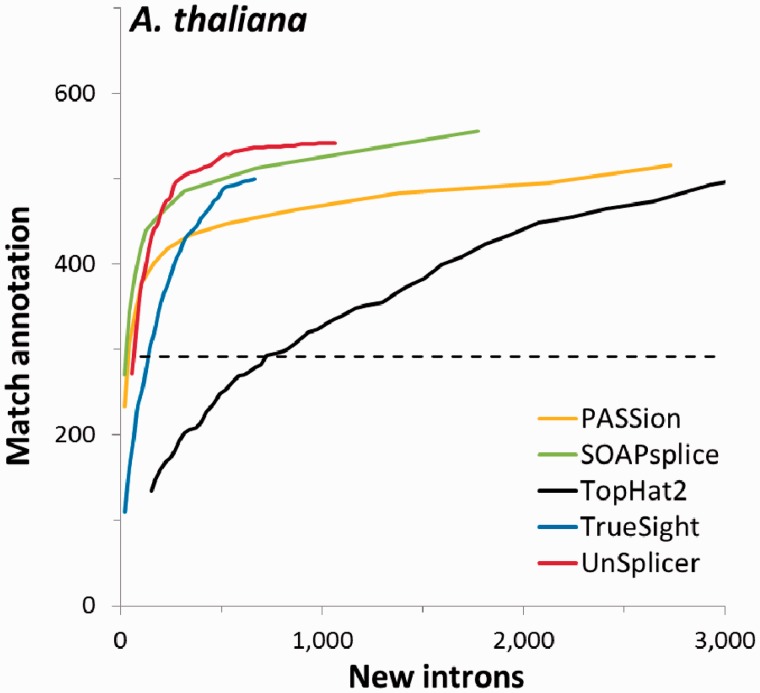
Counts of introns (matching annotation and new introns) with length >999 nt detected in read mapping from RNA-Seq data set SRR360205 to genome of *A. thaliana* by the five RNA-seq alignment tools.

Another interesting question was to find out how the programs’ accuracy in intron identification depends on intron length. We focused on long introns, ≥1000 nt, and built ROC type curves for this group of introns for five programs (Figure 7). At operating point T = 0.5, UnSplicer shows close performance to other programs (300 TPs and a few dozen FPs), while TopHat2, for the same number of TPs finds ∼900 more introns not matching annotation (Figure 7). Notably, with another (lower) value of threshold, UnSplicer finds 500 confirmed introns accompanied with 200 not matching annotation; for 500 confirmed long introns, other programs detect hundreds or even thousands of introns not matching annotation (Supplementary Figure S7). Comparison of length distributions of novel introns predicted in the *A. thaliana* genome by TopHat2 and UnSplicer (Supplementary Figure S7) substantiates the same point with more details.

### Mapping simulated RNA-Seq reads

**Figure 8.**
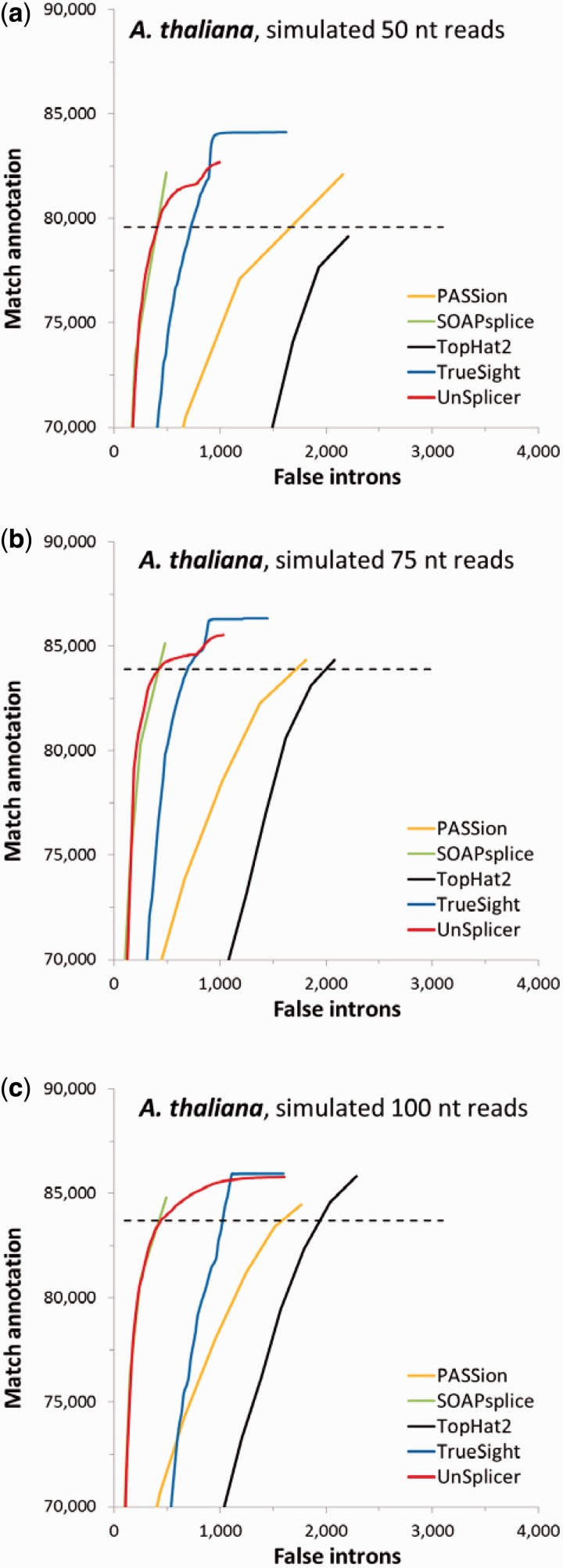
Performance of the five programs on simulated RNA-Seq data sets of *A. thaliana* (**a**) 50 nt; (**b**) 75 nt; (**c**) 100 nt. The curves represent nonnormalized ROC curves. The curves show much lower proportion of FP for a given total number of predictions in comparison with the real data set (Figure 6a).

We used Maq (1) to simulate RNA-Seq reads from 41 671 *A. thaliana* full-length cDNA sequences (with nucleotide modifications modeling sequencing errors with rate 0.02). We made three sets of reads, each containing 5 million paired-end sequences, with lengths 50, 75 and 100 nt. The cDNA sequences were used proportionally with the expression levels determined by TopHat2 and Cufflinks (18) for the RNA-Seq set SRR360205. The numbers of true-positive introns (matching annotation) and false-positive introns (absent in annotation) inferred in application of each program with a given threshold, are shown in Figure 8a–c by ROC-like curves. The ROC curves for UnSplicer and TrueSight were generated by changing the probabilistic score thresholds from 0 to 1 (as in Figure 6); the dashed lines corresponds to the use of the UnSplicer default threshold value 0.5. Curves for PASSion, SOAPsplice and TopHat2 where generated (similarly to the graphs in Figure 6) by use of SJ coverage as a threshold parameter. In mapping 100 nt long fragments (Figure 8c), the UnSplicer performance (and ROC curve) is closely matched by performances of SOAPsplice (for low threshold values) and TrueSight (for high threshold values). This tendency is also seen in Figure 8a–b for 50 and 75 nt long fragments. Notably, SOAPsplice marginally improves over UnSplicer at the intersection with the UnSplicer curve (T = 0.5) in all three cases (Figure 8a–c). On the other hand, when PASSion and TopHat2 generate the same number of TPs as UnSplicer (T = 0.5), they produce ∼1000 and ∼2000 more novel introns, respectively.

Notably, the relative numbers of novel introns identified by all five programs are significantly lower than ones we saw in mapping of real RNA-seq data sets. We assume that these false introns inferred in mapping of simulated reads, observed in relatively low number, are likely to be erroneously predicted due to the algorithm’s imperfection. We believe that the larger numbers of introns not matching annotation inferred in the experiments with real RNA-Seq data sets are due to additional ‘errors’ caused by noisy splicing producing nonfunctional transcripts.

By design of the experiment with simulated reads, the total number of reads aligned by each program could not exceed 10 million. TopHat2 and SOAPsplice aligned the largest numbers of reads (Supplementary Table S3), while TopHat2 predicted the largest number of SJs not confirmed by annotation.

Read coverage is an important factor in accurate intron (SJ) prediction. Interestingly, UnSplicer generates much lower number of novel introns uniformly with respect to the coverage, particularly in the low end (from 3 to 7) where TopHat2 generates more novel introns by thousands (Supplementary Figure S8). Large numbers of novel introns produced by TopHat2 could be related to processing of rather artifactual repeated reads that create high coverage for spurious SJs. Several programs are able to filter out such predictions; particularly, for this purpose, TrueSight and UnSplicer effectively use the entropy feature.

### Computational costs

All computational experiments were performed on 16 cores multiuser Linux system. Two measures of computational cost were reported: total elapsed time (‘wall clock’ time), and the CPU time for each program. Given that all five programs are multi-threaded, we specified eight threads use for each program. Computational costs for the three sets of *A. thaliana* simulated RNA-Seq reads alignments to genome (wall clock time and CPU time) are shown in two panels of Supplementary Table S4. In the bottom rows we show the running times of alignment of a real data set SRR360205. Because of the use of eight threads, the CPU time was often much greater than the wall clock time. The fastest program was TopHat2, with SOAPsplice in second place. For the set of 100 nt long reads, run times of UnSplicer and TrueSight were close to SOAPsplice. The most costly program to run was PASSion, which required 7–8 h of wall time to align the simulated reads, and over a day (34 h) to align the reads from SRR360205. UnSplicer and TrueSight were similar in speed. There are ∼4-fold more reads in SRR360205 compared with the simulated set, yet UnSplicer, TrueSight and TopHat2 each required less than four times the wall clock time to align the reads from SRR360205 compared with the set of 75 nt long simulated reads. PASSion required more than four times the wall time to align the same set of real reads in comparison with the same set of 75 nt long simulated reads.

## DISCUSSION

### Relationship to TrueSight

UnSplicer and TrueSight have three major differences: (i) in derivation of DNA sequence model parameters, (ii) in training set selection (for classification), (iii) in classification algorithm. UnSplicer derives model parameters by use of GeneMark-ES conducting unsupervised training on a reference genome, while TrueSight derives parameters directly from a set of SJs inferred by alignment with high confidence. In general, reliance on RNA-Seq alignments for deriving model parameters may lead to a bias in parameters of the model of protein-coding sequence due to overrepresentation of highly expressed genes in the RNA-seq population. It is well known that in prokaryotic and eukaryotic genomes, the codon usage pattern of highly expressed genes differs from codon usage patterns of genes expressed at moderate and low levels (28,29). UnSplicer’s use of GeneMark-ES tends to avoid this model bias problem by incorporating a large number (many thousands) of genes into self-training.

The training set for the UnSplicer classifier is compiled by a different set of heuristic rules (as described above), in comparison with rules used in TrueSight. The training set of positive examples in TrueSight was formed from the set of all SJs with (i) canonical splice sites, (ii) no mismatch errors in the alignment and (iii) confirmed by five or more alignments. The set of negative examples was taken from the set of gapped alignments spanning introns with (i) at least one noncanonical splice site, (ii) confirmed by only one alignment and (iii) the shorter overhang length close to the admitted minimum of 8 nt. The training set of positive examples was also used for deriving sequence model parameters. The UnSplicer rules for compiling SVM training sets were significantly different (see ‘Materials and Methods’ section).

The third major difference is in the design of the classification algorithm. TrueSight finds a decision boundary by the EM algorithm, maximizing the likelihood of all candidate SJs presented to the classifier. The EM algorithm searches for a hyperplane boundary separating positive and negative examples in the training set. UnSplicer uses the training set to find parameters of a Gaussian kernel SVM that maximizes agreement with the set of introns predicted *ab initio* in the development set.

In addition to the three major differences between UnSplicer and TrueSight, there are a few more minor differences. For instance, instead of the coding potential feature used by TrueSight for mapped SJs classification, UnSplicer uses two binary features, indicators of strand and frame concordance. These indicators are assigned to a value of zero for likely true introns (in protein-coding or UTR regions) and a value of one for likely FPs. Also, the two programs use different methods for assigning intron length score. TrueSight uses an intron length-related feature defined by the following rule: for an intron of length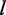, the score is zero if 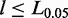, otherwise it is 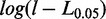, where 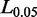 is the length >95% of candidate introns. In contrast, UnSplicer uses the gap length feature, a log likelihood of the event that a mapped intron has a given length; this feature is computed using the intron length distribution determined by GeneMark-ES. Earlier, we have demonstrated that intron length distributions derived by GeneMark-ES had a good fit to empirical distributions determined from transcript sequence alignments. For example, length distribution of introns inferred for the strawberry genome (*Fragaria vesca*) by the *ab initio* program GeneMark-ES (30) and length distributions of introns determined from transcript mapping to the same genome nearly coincide (Supplementary Figure S3). Finally, TrueSight uses splice site log likelihood scores summed into a single feature, while UnSplicer has two separate features for donor and acceptor sites.

Use of GeneMark-ES makes UnSplicer applicable to newly sequenced genomes complemented with newly sequenced RNA-Seq data as soon as the genome assembly and transcriptome sequencing is finished. No additional effort for obtaining any kind of manually curated training sets is necessary. Still, dependence of GeneMark-ES restricts UnSplicer to genomes where GeneMark-ES runs reliably, which largely includes compact genomes with homogeneous GC content. Fortunately, the vast majority of genomes of fungi, plants and animals, either already sequenced or in progress, belong to this category. In inhomogeneous genomes, such as large genomes of animals, model parameters needed for UnSplicer could be derived in conventional supervised manner.

Finally, we should note that TrueSight makes remapping and filtering steps after classification, while UnSplicer uses opposite order. The change of order in UnSplicer was made in consideration that remapping allows refining the alignment depth, which enters the classification algorithm as one of the features.

### Comparison of results of alignments of simulated and real RNA-Seq data

There is a noticeable difference in the results of RNA-Seq read mapping by each of five programs working with simulated and real RNA-seq reads. Much larger fractions of novel introns in the set of all predicted introns were predicted for real data set than for simulated ones. The difference is likely due to the presence of noisy splicing that made its way into the real RNA-seq reads. Notably, on simulated *A. thaliana* data, SOAPsplice and UnSplicer appeared to be comparable in terms of proportions of inferred introns that match and do not match the annotation. However, working with real *A. thaliana* RNA-Seq reads, UnSplicer predicted ∼10 000 fewer novel introns compared with SOAPsplice; at the same time UnSplicer mapped 33.7 million reads, while SOAPsplice mapped only 26.1 million.

Results of the tests on real RNA-Seq data (Figure 6) demonstrated that UnSplicer is more effective than other programs in inference of SJs (introns) that are likely to be functional.

We should emphasize that the suggested restriction of using UnSplicer for compact genomes (<500 Mb, which still covers vast majority of genomes sequenced to date) is related to the current version of GeneMark-ES. This version generates only one model for protein-coding region, which will not fit equally well to all the genes in a large genome with inhomogeneous GC content, such as mammalian genome with isochore organization. Current development of GeneMark-ES extension to inhomogenous genomes will make UnSplicer applicable to more complex genomes as well. The UnSplicer software is available for download from topaz.gatech.edu/GeneMark.

## SUPPLEMENTARY DATA

Supplementary Data are available at NAR Online.

Supplementary Data
