# Supplementary material for: UnSplicer: mapping spliced RNA-seq reads in compact genomes and filtering noisy splicing
Source: Nucleic Acids Res. 2013 Nov 19;42(4):e25. doi: 10.1093/nar/gkt1141 (PMC3936741; doi:10.1093/nar/gkt1141)
Supplement: Supplementary Data [file supp_42_4_e25__index.html]

UnSplicer: mapping spliced RNA-seq reads in compact genomes and filtering noisy splicing — UnSplicer: mapping spliced RNA-seq reads in compact genomes and filtering noisy splicing — Supplementary Data 

# UnSplicer: mapping spliced RNA-seq reads in compact genomes and filtering noisy splicing

## Supplementary Data

files

**Files in this Data Supplement:**

- Supplementary Data - docx file
