# Supplementary material for: UnSplicer: mapping spliced RNA-seq reads in compact genomes and filtering noisy splicing
Source: Nucleic Acids Res. 2013 Nov 19;42(4):e25. doi: 10.1093/nar/gkt1141 (PMC3936741; doi:10.1093/nar/gkt1141)
Supplement: Supplementary Data [file supp_gkt1141_nar-01333-met-n-2013-File010.docx]

**Supplementary Materials**

**UnSplicer: Mapping Spliced RNA-Seq Reads in Compact Genomes and Filtering Noisy Splicing**

Paul D. Burns, Yang Li, Jian Ma and Mark Borodovsky

**Outline**

1. Features used for candidate splice junction (SJ) classification
2. Training the radial basis function (RBF) support vector machine (SVM)
3. Additional performance assessment

**Features used for candidate splice junction classification**

UnSplicer defines nine features for each candidate SJ: five alignment derived features, and four sequence derived features. Some of the features were described in the TrueSight publication (4).

*Alignment depth score*

The alignment depth or coverage, D, is the number of RNA-Seq reads aligned across a given SJ. This number includes the reads aligned across the SJ in the realignment step, with the initially predicted intron spliced out of the “pseudo-transcript”. The alignment depth score is equal to
log (D).

*Max shorter overhang length*

If a read of length L is aligned across a SJ so that n nucleotides are aligned upstream to the SJ and L-n downstream, then the length of *shorter* “overhang” is $min\left[ n,L-n \right].$ We want to single out potentially less reliable SJs with consistently short *shorter* overhangs regardless of the side. Note, that the algorithm does not allow overhangs shorter than 8 nt. Let say a SJ has D alignments in the second step of alignment (including re-aligned reads), with overhang lengths for alignment *i, -* $n_{i}$ upstream and $L-n_{i},$ downstream from the SJ - with $i=\left\{ 1,\ldots,D \right\}$. We define the max *shorter* overhang length as

overhang =${max}_{i}\left( min\left( n_{i},L-n_{i} \right) \right)$.

The final value of the *shorter overhang score* is defined as:

overhang score = overhang-8

*Entropy*

Consider an RNA-Seq read of length *n* spanning a SJ, with *n* and *L-n* nucleotides on either side of the SJ. We use the read position index *i* indicating the SJ location so *n=i* (see Fig. S1). For a group of $D$ reads aligned across the same SJ, we construct an empirical distribution of frequencies $f_{i}$ such that$f_{i}=m_{i}/D$, where $m_{i}$ is the number of reads split at position $i$. The *entropy score* is then determined by

$$H=\sum_{i=1}^{L} f_{i}logf_{i}$$

Genomic sequence

Putative intron

boundaries (p,q)

**Read position i**

p

q

**Figure S1.** Alignment of an RNA-Seq read of length $L$ to genomic DNA is split over an intron connecting two exons. The read has a SJ at positions *i* and *i+1*, where $1\leq i<L$. The nucleotide in position *i* is aligned to nucleotide in position *p-1* in genome and, respectively, the read nucleotide in position *i+1* is aligned to the nucleotide in genomic position *q+1*.

*Coverage skew*

The coverage skew score depends on the number of ungapped alignments on the flanks of a spliced intron (p,q) and the number of ungapped alignments that fall into the putative intron (Fig. S2). We determine counts of ungapped read alignments that fall into four intervals: intervals upstream to (Ap) and downstream from (Bp) position p (the 5’ end of putative intron), and intervals upstream to (Bq) and downstream from (Aq) position q (the 3’ end of putative intron).

For a true SJ, it is expected that the flanks will get more RNA-Seq coverage than the interior, the intron. Therefore, the quantities Ap-Bp and Aq-Bq should be positive and their sum, the skew score, will be positive for nearly all true splice junctions. Since most of false positives have a negative skew score (see Fig. 3) this feature is useful for selecting a negative set of SJs (introns).

**Figure S2.** Schematics of computation of the coverage skew score

*Gap (intron) length*

The intron length score is the log of frequency of introns having given length. The frequency distribution of intron length is determined from the set of introns predicted by GeneMark-ES in the final prediction step (after generating model parameters in the last iteration of unsupervised training). A uniform kernel is used to get a smooth distribution function.

Earlier we have observed that the intron length distribution derived from the EST gapped alignments is close to the length distribution derived by GeneMark-ES (e.g. Fig. S3 shows the intron length distributions for the strawberry genome, *Fragaria vesca*).

**Figure S3**. Comparison of strawberry (*F. vesca*) intron length distributions determined by GeneMark-ES (blue) and one derived from alignment of EST sequences (red).

*Donor and acceptor splice site scores*

The scores of donor and acceptor sites were computed based on position frequency models of splice site sequences $P_{i}\left( x_{i} \right)$and background sequences $Q\left( x_{i} \right)$. Parameters of the site model $P_{i}^{j}$, are either nucleotide positional probabilities (for the zero order model, j = 0), or Markov transition probabilities (for the first order model, j = 1). The score was defined as a log likelihood ratio, i.e. the log of the ratio of probability that given sequence S, of length N, is a splice site to the probability that sequence S is not a splice site. For instance, the splice site score for zero-order models can be written as

$$W=\sum_{i=1}^{N} log\frac{P_{i}^{0}\left( x_{i} \right)}{Q^{0}\left( x_{i} \right)}$$

where $N$ is the width of the site and $i$ is the sequence position. Note that the splice site canonical dinucleotides do not contribute to the score of either the donor or acceptor since the same dinucleotides are present in the sample of background sequences.

The order of the site model is selected depending on the size of training set - the number of splice sites detected by GeneMark-ES. Normally, the training set size is sufficient for reliable derivation of parameters of the first order model.

*Strand concordance indicator*

The strand concordance indicator (SCI) is a binary numerical feature which equals to 1 if strand concordance is not detected (when predicted intron belongs to an opposite strand to an *ab initio* predicted gene) and 0 otherwise. SCI value is assigned to 1 if the three conditions are satisfied:
i/ the SJ strand can be identified based on intron terminal dinucleotide sequences GT-AG, GC-AG, or AT-AC; ii/ either p or q, the intron border positions in genomic DNA lie inside a predicted gene (between the positions of the first nucleotide of a predicted start codon and the last nucleotide of the stop codon); iii/ the inferred strand of the SJ is opposite to the strand of predicted gene overlapping with predicted intron (p, q). If one of the three conditions is not fulfilled, SCI is equal to 0. The assignments of SCI features (as well as the frameshift indicator features) are illustrated in Fig. S4.

*Frame concordance (frameshift) indicator*

The frame concordance indicator is a binary numerical feature similar to SCI. This feature is equal to 1 if an intron splicing (SJ) leads to a frameshift in a gene. Specifically, it is equal to 1 if i/ for the inferred intron (p,q) *both* positions p and q lie inside a GeneMark-ES predicted gene (delimited by the first nucleotide of a start codon, and the last nucleotide of the stop codon); ii/ the upstream and downstream regions around the intron (p,q) are predicted as coding exons; iii/ the GeneMark-ES predicted reading frames in these exons are not in agreement after splicing out intron (p,q). The frame concordance (frameshift) indicator is assigned to 1 if all three conditions hold, otherwise to 0.

**Figure S4.** Frameshift and strand concordance features: a/ the candidate SJ agrees with an *ab initio* predicted intron, both indicators are equal to 0; b/ one boundary falls into the interior of a predicted coding exon, a frameshift will occur if the interval length L is not a multiple of three, c/ same as in (b), except that the intron is located in the opposite strand to the predicted gene, therefore the strand concordance feature is equal to 1; d/ the intron includes predicted exon of length M, resulting in a frame shift if M is not a multiple of 3; e/ both indicators are 0 because the predicted intron does not overlap with a predicted gene.

**Training of the radial basis function support vector machine (RBS SVM)**

Selection of a training set for candidate SJs classification is a task that has to balance somewhat competing objectives. First, the training set has to be correctly labeled. Second, it is beneficial to have a large set of data representing without bias distributions of features values in positive and negative candidate SJs. To bypass the need in an expert we used as training sets the subsets of SJs that could be reliably labeled (positive or negative) based on the alignment features. We used three of the nine SJ features for the training set selection; the remaining six features were used to classify the SJs in the training set. Notably, all training was performed on canonical SJs.

Training sets of positive examples for all four genomes were compiled from SJs having *both* an entropy score larger than 20, and a shorter overhang score larger than 20 (see Fig. 3 for *D. melanogaster* in the main text). For reads shorter than 60 nt, the overhang threshold was determined by expression$0.5\times\left( D+9 \right)$, where $D$ is the half length of a read. This heuristic rule helped maintain a reasonably large number of positive examples in the training set of short reads.

Training sets of negative examples for all four genomes were compiled based on the observation that negative SJs are highly enriched i/ among SJs with skew scores less than -1, as well as ii/ among SJs with shorter overhang score less than or equal to 2 (see Fig. 4 for *A. thaliana* in the main text). An SJ was labeled as a negative if either condition is true.

The whole training set was selected to be balanced, with equal numbers of positive and negative examples. Also, the size of the training set was limited by 10,000 but could be smaller if the number of available positive or negative examples obtained by this procedure was less than 5,000.

Thus compiled training set was used to define a decision boundary in the 6-dimensional space of all other features. The program LIBSVM (6) was used for training the SVM and subsequent classification of SJs.

The decision boundary in the SVM method was found for the Gaussian kernels with two parameters: scalar $\gamma$ ($\gamma=1/\sigma^{2}$), and an error cost c. We compiled additional set of 10,000 SJs (not overlapping with the training set) to serve as the development set (see main text). We defined labels of these SJs (positive and negative) with respect to the agreement with the GeneMark-ES intron predictions. Then to find the best values ($c, \gamma)$ we used a grid of pairs ($c, \gamma)$as the set of parameters for the Gaussian kernels and trained SVM for each pair ($c, \gamma)$ on the training set; then we looked for a maximum value X = TP-FP (the difference between numbers of “correctly classified” and “incorrectly classified”) on the grid.

A grid search for the *A. thaliana* RNA-seq data is illustrated in Fig. S5 (a-d) with the best pair of values of ($logc, log\gamma)$ found to be (-7, -2). It was shown that the vector of optimal parameters of SVMs was located on the deep red “ridge” of the ($logc, log\gamma)$ heat map (Fig. S5c) showing values X (17). Moreover, the pattern of change of X depending on ($\gamma,c)$ in all four data sets related to the four genomes is similar (Fig. S6). Regions similarly situated in $(logc, log\gamma)$ parameter plane (panels *a-d* of Fig. S6) result in under-fitting (the development set SJs were labeled the same way for such ($c, \gamma)$), or over-fitting (for such ($c, \gamma)$all the development set SJs were labeled as false unless they lie very close to a positive training sample). A zone of ($c, \gamma)$ with good performance was situated on a ridge starting from roughly $(logc, log\gamma)=(-7, 0,)$ and extending with a slope of -1. Such a trend was reported to be a general property of RBF SVMs (31). To reduce computational costs, taking to account this pattern, the grid search was restricted to a vicinity of the ridge. The decision boundary became closely approximating a hyperplane, as $c$ increased and $\gamma$ decreased along the ridge. Still, we observed, most distinctly in *C. elegans*, that the best performance was achieved at a boundary different from a hyperplane, specifically in the region near $(logc, log\gamma)=(-6, 0)$. This finding demonstrated that the RBF SVM improved performance compared with a hyperplane boundary.

After the optimal parameters ($c, \gamma)$ were found by the grid search, all unlabeled candidate canonical SJs were classified by the SVM algorithm. After classification of canonical SJs, prediction of non-canonical SJs was performed using the same decision boundary.

**Figure S5.** Performance of SVM kernels with various values of ($c, \gamma)$ on a development set of SJs inferred from mapping the RNA-Seq set SRR360205 to genome of *A. thaliana*. Panels: a: number of SJs in development set for which classifier label “agrees” with the GeneMark-ES label; b: number of SJs for which classifier label “does not agree” with the GeneMark-ES label; c: the difference of numbers in panel *a* minus those in *b*; d: the ratio of values in panel *a* to those in *b*. In this example, the best pair ($c, \gamma)$ in terms of panel *c* is such that log(*c*) = -7, and log($\gamma$) = -2.

**Figure S6.** We observed that for all examined RNA-seq data sets, regions similarly situated in panels *a,b,c,d* in the $(logc, log\gamma)$ planes resulted in either under-fitting or over-fitting the training data. It was also observed that ($c, \gamma)$ vectors from a similarly situated ridge (a zone of highest values of the X function) produce the best overall performance in each case.

**Additional Tables and Figures**

**Table S1**. The numbers of annotated splice junctions in *A. thaliana* detected by aligning the RNA-Seq data set SRR360205. Splice junctions connecting coding exons are labeled as ‘coding’ and all others ‘non-coding’. The ratio of the number of confirmed annotated coding SJs to the number of confirmed non-coding SJs is shown in the bottom row. We used the three sets of UnSplicer predictions, with the thresholds: T= 0.1, 0.5, 0.9. TopHat2 was used with max intron length 10k. Other programs were used with default settings.

| SJ type | UnSplicer (T=0.1) | UnSplicer (T=0.5) | UnSplicer (T=0.9) | TrueSight | TopHat2 | SOAPsplice | PASSion |
| --- | --- | --- | --- | --- | --- | --- | --- |
| coding | 92,306 | 88,980 | 70,902 | 92,977 | 92,271 | 94,207 | 93,934 |
| non-coding | 5,737 | 5,073 | 3,331 | 6,074 | 6,003 | 6,218 | 5,236 |
| coding / non-coding | 16.09 | 17.54 | 21.29 | 15.31 | 15.37 | 15.15 | 17.94 |

**Table S2**. The distribution of the numbers of short exons predicted in *A. thaliana* by aligning the RNA-Seq data set SRR360205. Likely TP agree with the genome annotation, while likely FP are not found in the annotation. Since the reads have length 76nt the number of detected exons longer than 70nt gets is sharply down in the last length bin. SOAPsplice does not align reads with more than one gap therefore we excluded this program as well as TopHat2 since the microexon information is not readily available in the program output. We used the three sets of UnSplicer predictions, with the thresholds: T= 0.1, 0.5, 0.9. Other programs were used with default settings.

| **Exon length (nt)** | **UnSplicer (T=0.1)** | **UnSplicer (T=0.5)** | **UnSplicer (T=0.9)** | **TrueSight** | **PASSion** |
| --- | --- | --- | --- | --- | --- |
|  | likely TP / likely FP | likely TP / likely FP | likely TP / likely FP | likely TP / likely FP | likely TP / likely FP |
| **0-9** | 3 / 5 | 3 / 4 | 3 / 3 | 2 / 2 | 3 / 101 |
| **10-19** | 50 / 32 | 47 / 16 | 45 / 5 | 41 / 10 | 70 / 3,627 |
| **20-29** | 325 / 101 | 311 / 65 | 270 / 34 | 315 / 91 | 357 / 5,037 |
| **30-39** | 1,058 / 189 | 1,030 / 100 | 842 / 45 | 1,030 / 239 | 1,009 / 4,059 |
| **40-49** | 2,470 / 234 | 2,397 / 155 | 1,973 / 78 | 2,351 / 288 | 2,151 / 2,496 |
| **50-59** | 3,611 / 256 | 3,532 / 199 | 2,936 / 129 | 3,598 / 274 | 2,405 / 1,009 |
| **60-69** | 4,946 / 222 | 4,889 / 187 | 4,344 / 141 | 5,082 / 233 | 260 / 36 |
| **70-79** | 1,207 / 41 | 1,267 / 37 | 1,212 / 35 | 1,192 / 33 | 0 / 0 |
| **likely TP (total)** | 13,733 | 13,476 | 13,625 | 13,611 | 6,255 |
| **likely FP (total)** | 1080 | 763 | 470 | 1,170 | 16,365 |
| **Sp (%)** | 92.8 | 94.6 | 96.1 | 92.1 | 27.7 |

**Table S3**. The numbers of 10 mln simulated reads (of the three read lengths) aligned by each program (in millions of reads) to the *A. thaliana* genome.

|  | **UnSplicer** | **TrueSight** | **TopHat2** | **SOAPsplice** | **PASSion** |
| --- | --- | --- | --- | --- | --- |
| 50 nt | 9.560 | 9.394 | 9.196 | **9.599** | 8.896 |
| 75 nt | 8.017 | 7.924 | 7.878 | **8.422** | 8.299 |
| 100 nt | 6.953 | 6.873 | 7.243 | 7.424 | **7.790** |

**Table S4**. The running times, shown in (hours : minutes), needed by each of the five programs to map 10 mln of simulated reads to the *A. thaliana* reference genome. The bottom row indicates the time required to align the data set SRR360205 comprised of 20.9 million paired-end reads with length 76 nt to the *A. thaliana* reference genome.

| Wall clock (h:m) | **UnSplicer** | **TrueSight** | **TopHat2** | **SOAPsplice** | **PASSion** |
| --- | --- | --- | --- | --- | --- |
| 50 nt | 1:29 | 1:13 | 0:22 | 0:15 | 7:54 |
| 75 nt | 1:39 | 1:31 | 0:19 | 0:45 | 8:43 |
| 100 nt | 2:08 | 1:55 | 0:28 | 1:45 | 7:34 |
| SRR360205 (76 nt) | 5:37 | 5:19 | 1:10 | 3:55 | 34:29 |
|  |  |  |  |  |  |
| CPU time (h:m) | **UnSplicer** | **TrueSight** | **TopHat2** | **SOAPsplice** | **PASSion** |
| 50 nt | 3:51 | 3:09 | 0:51 | 0:50 | 10:18 |
| 75 nt | 3:27 | 4:00 | 0:58 | 2:55 | 15:06 |
| 100 nt | 4:36 | 4:53 | 1:29 | 5:28 | 22:02 |
| SRR360205 (76 nt) | 13:23 | 19:49 | 6:26 | 23:35 | 102:42 |
|  |  |  |  |  |  |


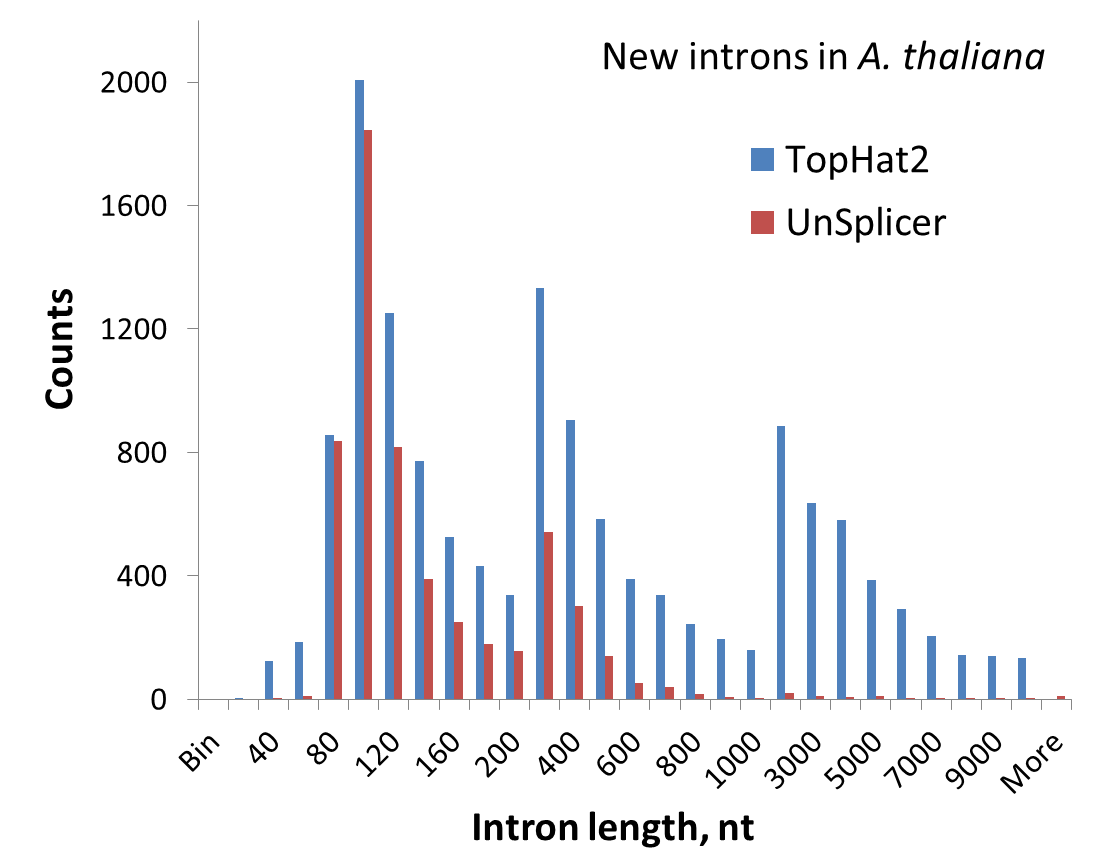


**Figure S7**. Length distribution of the novel introns identified by UnSplicer and TopHat2 in SRR360205 RNA-Seq dataset of *A. thaliana*. Both program runs with max intron length 10k. TopHat2 predicts significantly more novel introns than UnSplicer as intron length increases. **Note that the scale of X axis is not uniform.**

Figures S7 and S8 are made for conditions/parameters when TopHat2 and UnSplicer identify the same number of introns matching annotation. This number is close to 94,000 for UnSplicer running with threshold 0.5 and for TopHat2 selecting all introns identified by minimum three spanning reads.

Numbers of observed new introns in the range of 60 to 120 nt are similar for both algorithms. The location of major peak matches the peak of true intron length distribution in *A. thaliana*. Significant difference between two programs is observed for long introns. This difference is making two orders of magnitude for introns longer than 1,000 nt.


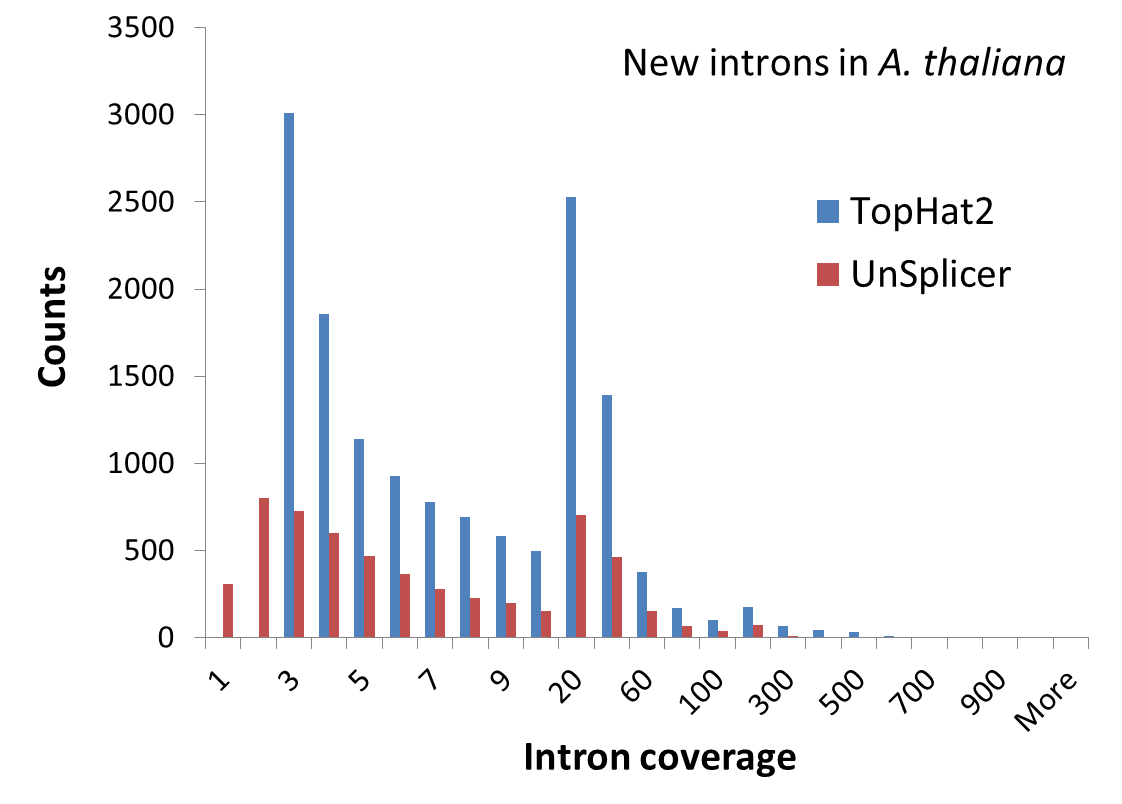


**Figure S8.** Distribution of read coverage of novel splice junctions (introns) identified by UnSplicer and TopHat2 in SRR360205 RNA-Seq dataset of *A. thaliana*. Both program runs with max intron length 10k. TopHat2 makes significantly more novel predictions with counts decreasing exponentially as the coverage increases (**The scale of X axis is non-uniform**). Note that introns mapped by TopHat2 with coverage less than 3 are discarded in this comparison.

The reduction in novel SJs (introns) generated by UnSplicer is related to the filtering of SJ with low probabilistic scores. For instance, UnSplicer gives low score to some splice junctions with high coverage due to the effect of entropy score which discriminates against SJs with a large number of identical reads aligned across the SJ.


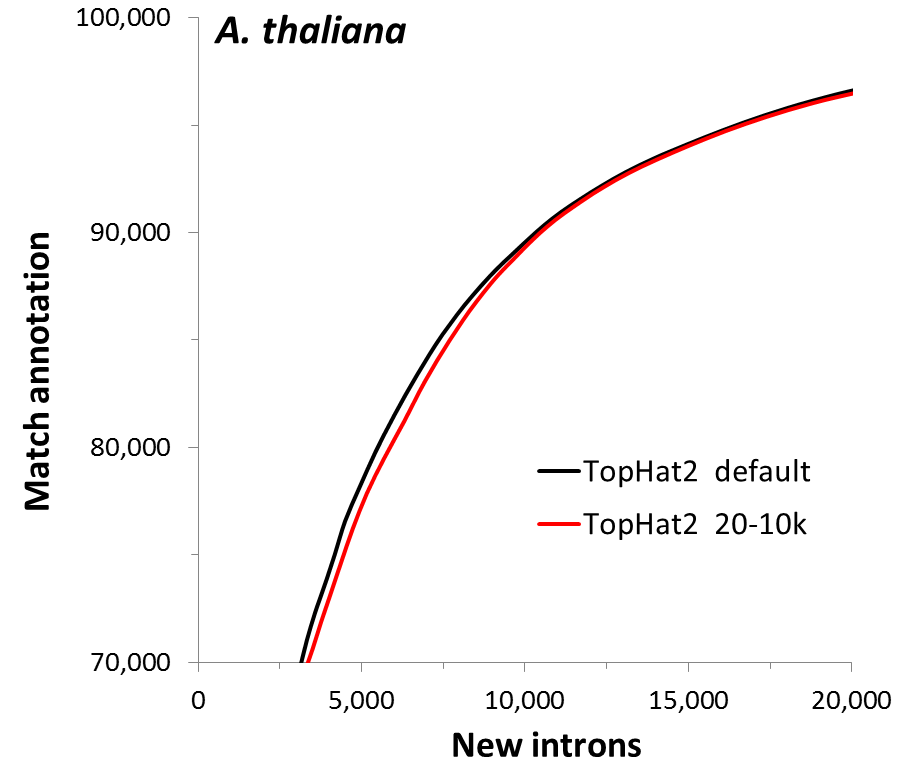


**Figure S9.**  Additional comparison of the runs of TopHat2 with 500k and 10k max intron length and min intron length 20 nt. Change of restrictions on the intron length did not produce significant change in the shape of the curve (see also Fig. 6a)
